# Intensive care of traumatic brain injury and aneurysmal subarachnoid hemorrhage in Helsinki during the Covid-19 pandemic

**DOI:** 10.1007/s00701-020-04583-4

**Published:** 2020-09-24

**Authors:** Teemu Luostarinen, Jyri Virta, Jarno Satopää, Minna Bäcklund, Riku Kivisaari, Miikka Korja, Rahul Raj

**Affiliations:** 1 Division of Anaesthesiology, Department of Anaesthesiology, Intensive Care and Pain Medicine, Helsinki University Hospital, Topeliuksenkatu 5, PO BOX 266, 00029 HUS Helsinki, Finland; 2 Department of Neurosurgery, University of Helsinki and Helsinki University Hospital, Helsinki, Finland; 3 Division of Intensive Care, Department of Anaesthesiology, Intensive Care and Pain Medicine, Helsinki University Hospital, Helsinki, Finland

**Keywords:** COVID-19, Traumatic brain injury, Subarachnoid hemorrhage, Intensive care

## Abstract

**Background:**

To ensure adequate intensive care unit (ICU) capacity for SARS-CoV-2 patients, elective neurosurgery and neurosurgical ICU capacity were reduced. Further, the Finnish government enforced strict restrictions to reduce the spread. Our objective was to assess changes in ICU admissions and prognosis of traumatic brain injury (TBI) and aneurysmal subarachnoid hemorrhage (SAH) during the Covid-19 pandemic.

**Methods:**

Retrospective review of all consecutive patients with TBI and aneurysmal SAH admitted to the neurosurgical ICU in Helsinki from January to May of 2019 and the same months of 2020. The pre-pandemic time was defined as weeks 1–11, and the pandemic time was defined as weeks 12–22. The number of admissions and standardized mortality rates (SMRs) were compared to assess the effect of the Covid-19 pandemic on these. Standardized mortality rates were adjusted for case mix.

**Results:**

Two hundred twenty-four patients were included (TBI *n* = 123, SAH *n* = 101). There were no notable differences in case mix between TBI and SAH patients admitted during the Covid-19 pandemic compared with before the pandemic. No notable difference in TBI or SAH ICU admissions during the pandemic was noted in comparison with early 2020 or 2019. SMRs were no higher during the pandemic than before.

**Conclusion:**

In the area of Helsinki, Finland, there were no changes in the number of ICU admissions or in prognosis of patients with TBI or SAH during the Covid-19 pandemic.

## Introduction

The Covid-19 pandemic, caused by severe acute respiratory syndrome coronavirus 2 (SARS-CoV-2), prompted unprecedented restriction measures to prevent the spread of the virus. In hospitals, major reorganizations, including reschooling of healthcare professionals and reallocation of medical supplies, took place to be able to tackle the expected sharp rise in SARS-CoV-2 infected patients. Also, neurosurgery was affected, as non-emergent surgeries were postponed, and outpatient clinic visits cancelled [19]. Accordingly, there were concerns that the diagnosis and treatment of other illnesses requiring intensive care might suffer due to the sudden and massive reallocation in intensive care resources [16]. However, a previous European survey study suggested a lower number of treated aneurysmal subarachnoid hemorrhage (SAH) and traumatic brain injury (TBI) patients during the pandemic [11]. Yet, a study from Charité, Berlin, did not find any change in the number of aneurysmal SAH or TBI admissions compared previous years.

In Finland, the first wave of Covid-19 hit the Helsinki area the worst. Thus, we saw the closing of borders, restrictions of free movement, and the closing of schools and restaurants under the enforcement of the Emergency Powers Act. Consequently, the incidence of SARS-CoV-2 infection and, thus its imposed burden on the healthcare system, remained relatively low and our capacity to care for emergency neurosurgical cases remained good.

Our aim was to give a detailed description regarding admissions and outcomes of patients with TBI and aneurysmal SAH treated in the neurosurgical ICU in Helsinki during the Covid-19 pandemic. Further, we report changes in emergency and non-emergency neurosurgical operations during this period. We hypothesized a decrease in the occurrence of TBIs due to strict regulations limiting movement and travel in the region but a similar occurrence of aneurysmal SAH compared with the previous year.

## Methods

We reviewed all consecutive cases admitted due to aneurysmal SAH or TBI to the neurosurgical ICU of Helsinki University Hospital between 1 January and 31 May 2020 and between 1 January and 31 May 2019. The 2019 period was used as a reference for 2020. As of 2019, the neurosurgical department in Helsinki covers a population of approximately 2.2 million people, of which 1.8 million (82%) are 18 years or older (the catchment area expanded between 2018 and 2019). We included only adults (18 years or older), as children are treated at a different location (Children’s Hospital, Helsinki). In the region of Helsinki and Uusimaa (referred to as the HUS area), treatment of TBIs and SAHs that require intensive care has for decades been centralized at the neurosurgical department in Helsinki. Essentially, all patients with aneurysmal SAH admitted to the hospital are transferred to our unit (with the possible exception of patients first admitted to other hospitals that are deemed to have a dire prognosis not benefitting from neurosurgical or neurointensive care) [7]. Regarding TBIs, patients with severe TBI and those requiring neurosurgical care are transferred to our unit [10]. Patients with moderate-to-mild TBI not requiring neurosurgical or neurointensive care are not regularly admitted to our unit and may be treated at local hospitals according to ability and capacity.

### Major Covid-19 restrictions in Finland

As of 17 March 2020 (week 12), the Emergency Powers Act was enforced in Finland. To prevent the spread of SARS-CoV-2, between 27 March (end of week 13) and 15 April (middle of week 16) 2020, travel in and out of the region of Helsinki and Uusimaa (population 1.7 million) was prohibited (with the exception of hospital transfers and work- or family-related travel; i.e., the restrictions applied mainly to leisure-related travel). Of importance, hospitals situated outside the Uusimaa border but still belonging to the catchment area of Helsinki University Hospital transferred patients requiring neurointensive or neurosurgical care normally. From 3 April (end of week 14) to 31 May (end of week 22), all restaurants, cafes, and bars were closed. From 18 March (middle of week 12) to 14 May (middle of week 20), schools were closed

At the end of March, the Joint Authority of the Helsinki and Uusimaa Hospital District started to decrease non-urgent treatment with the goal of doubling current ICU capacity to treat SARS-CoV-2 patients.

### Statistical analyses

The frequency of TBI and aneurysmal SAH admissions (separately and jointly) are presented in a stepwise fashion, increasing every week, and as the raw number of admissions every 2 weeks. The number of emergency operations is shown on a weekly basis. The number of SARS-CoV-2 treated in the ICUs in HUS was reported twice a week and is shown as the cumulative number of patients receiving intensive care at that time.

Week 12 was defined as the cut-off, as extended measures for stopping the spread were implemented following the enforcement of the Emergency Powers Act. Thus, we divided the patients into two intervals—the early interval includes weeks 1–11 (in 2019, 1 January to 17 March; in 2020, 1 January to 15 March) and the late interval includes weeks 12–22 (in 2019, 18 March to 31 May; in 2020, 16 March to 31 May).

We report the number of admitted TBI and SAH patients tested for SARS-CoV-2 in 2020. The test was carried out with most unconscious patients whose background information could not rule out SARS-CoV-2.

We used the 30-day all-cause mortality as the primary patient outcome of interest. We assessed standardized mortality rates (SMR) for each month and interval. The predicted risk of 30-day mortality was calculated using multivariable logistic regression, accounting for age (as a continuous variable), Glasgow Coma Scale (GCS as a continuous variable, also presented as categories 3–8, 9–12, and 13–15) score on admission (or last reliable assessment before intubation and sedation), and admission pupillary light reactivity (categories: normal, abnormal). The predicted risk-of-death model was based upon patients admitted in 2019 and tested on patients admitted in 2020. The performance of the model is given as the area under the curve (AUC) of the receiver operating characteristic with 95% confidence interval (CI). SMRs are compared using a *t* test.

All neurosurgical non-emergency and emergency surgeries and neurosurgical endovascular procedures performed during the same periods were anonymously extracted by a surgical manager software program (Centricity™ Opera, GE Healthcare, Chicago, Ill, USA) and the Picture Archiving and Communication System software (Siemens Syngo, Siemens Healthineers, Erlangen, Germany).

The study is part of the ongoing quality control project of brain injury patients treated at our neurosurgical intensive care unit. The research committee of Helsinki University Hospital approved our quality control project and waived the need for informed consent (HUS/26/2018 §134).

## Results

The total study population consisted of 224 patients (105 admitted in 2019 and 119 admitted in 2020). Patient characteristics according to admission year are shown in Table 1. Of all patients, 123 (55%) were TBI patients and 101 (45%) were SAH patients. There were no notable differences in diagnoses (TBI vs. SAH), interval (early vs. late), age, sex, GCS score, pupillary light reaction, and ICU length-of-stay between the 2019 and the 2020 admissions. There were no notable differences in patient characteristics for TBI or SAH patients admitted in 2019 and 2020 (Table 2). For SAH patients, there were no differences between those admitted in the early and late intervals in 2020 (Table 3). TBI patients admitted in the late interval in 2020 underwent craniotomy or decompressive craniectomy less frequently than patients admitted in the early interval (61 vs. 35%, *p* = 0.038). In total, 24 patients (11 TBI and 13 SAH patients) were tested for SARS-CoV-2, none being positive.

**Table 1:** Patient characteristics per year of admission

Variable	2019 (*n* = 105)	2020 (*n* = 119)	*p* value
Diagnosis			
TBI	56 (53%)	67 (56%)	0.656
SAH	49 (47%)	52 (44%)	
Interval			
Early	53 (50%)	61 (51%)	0.907
Late	52 (50%)	58 (49%)	
Age (median (IQR))	50 (62, 72)	56 (44, 71)	0.163
Female (*n* (%))	44 (42%)	45 (38%)	0.533
GCS score (*n* (%))			
3–8	39 (37%)	37 (31%)	0.480
9–12	18 (17%)	18 (15%)	
13–15	48 (46%)	64 (54%)	
Pupillary light reaction (*n* (%))			
Normal	87 (83%)	95 (80%)	0.563
Abnormal	18 (17%)	24 (20%)	
Days in ICU (median (IQR))	5 (3, 9)	5 (2, 12)	0.988
SARS-CoV-2 tested^a^ (*n* (%))	NA	24 (20%)	NA
SARS-CoV-2 positive^b^ (*n* (%))	NA	0 (0%)	NA

Early interval includes weeks 1 to 11 and late interval includes weeks 12 to 22

Abbreviation: *GCS*, Glasgow Coma Scale; *ICU*, intensive care unit; *TBI*, traumatic brain injury; *SAH*, aneurysmal subarachnoid hemorrhage

^a^Tested during the initial admission

^b^Of tested

**Table 2:** Patient characteristics per disease group and year of admission

Variable	Disease group					
	TBI (*n* = 123)			SAH (*n* = 101)		
	2019 (*n* = 56)	2020 (*n* = 67)	*p* value	2019 (*n* = 49)	2020 (*n* = 52)	*p* value
Interval						
Early	31 (55%)	33 (49%)	0.500	22 (45%)	28 (54%)	0.369
Late	25 (45%)	34 (51%)		27 (55%)	24 (26%)	
Age (median (IQR))	63 (54, 72)	60 (41, 72)	0.416	58 (49, 70)	55 (48, 67)	0.394
Female (*n* (%))	13 (23%)	13 (19%)	0.606	31 (63%)	32 (62%)	0.858
GCS score (*n* (%))						
3–8	22 (39%)	21 (31%)	0.402	17 (35%)	16 (31%)	0.901
9–12	14 (25%)	14 (21%)		4 (8%)	4 (8%)	
13–15	20 (36%)	32 (48%)		28 (57%)	32 (61%)	
Pupillary light reaction (*n* (%))						
Normal	45 (80%)	58 (87%)	0.353	42 (86%)	37 (71%)	0.076
Abnormal	11 820%)	9 (13%)		7 (14%)	15 (29%)	
TBI operative treatment						
Craniotomy or decompressive craniectomy	31 (55%)	32 (47%)	0.401	NA	NA	NA
Aneurysm treatment						
Endovascular	NA	NA	NA	26 (53%)	38 (73%)	0.111
Microsurgical	NA	NA	NA	14 (29%)	9 (17%)	
No treatment^a^	NA	NA	NA	9 (18%)	5 (10%)	
Days in ICU (median (IQR))	4 (2, 7)	3 (2, 8)	0.869	8 (4, 14)	9 (3, 13)	0.923
SARS-CoV-2 tested^b^ (*n* (%))	NA	11 (16%)	NA	NA	13 (25%)	NA
SARS-CoV-2 positive^c^ (*n* (%))	NA	0 (0%)	NA	NA	0 (0%)	NA

Abbreviation: *GCS*, Glasgow Coma Scale; *TBI*, traumatic brain injury; *SAH*, aneurysmal subarachnoid hemorrhage

^a^In all cases aneurysm treatment was withheld due to dismal prognosis

^b^Tested during the initial admission

^c^Of SARS-CoV-2 tested

**Table 3:** Patient characteristics per disease group according to 2020 time epoch

Variable	Disease group					
	TBI (*n* = 67)			SAH (*n* = 52)		
	Early (*n* = 33)	Late (*n* = 34)	*p* value	Early (*n* = 28)	Late (*n* = 24)	*p* value
Age (median (IQR))	59 (43, 75)	62 (38, 71)	0.906	55 (46, 68)	55 (51, 65)	0.838
Female (*n* (%))	7 (21%)	6 (18%)	0.712	14 (50%)	18 (75%)	0.065
GCS score (*n* (%))						
3–8	9 (27%)	12 (35%)	0.175	9 (32%)	7 (29%)	0.967
9–12	10 (30%)	4 (12%)		2 (7%)	2 (8%)	
13–15	14 (43%)	18 (53%)		17 (61%)	15 (63%)	
Pupillary light reaction (*n* (%))						
Normal	30 (91%)	28 (82%)	0.305	18 (64%)	19 (79%)	0.238
Abnormal	3 (9%)	6 (18%)		10 (36%)	5 (21%)	
TBI operative treatment						
Craniotomy or decompressive craniectomy	20 (61%)	12 (35%)	0.038	NA	NA	NA
Aneurysm treatment						
Endovascular	NA	NA	NA	20 (72%)	18 (74%)	0.605
Microsurgical	NA	NA	NA	6 (21%)	3 (13%)	
No treatment^a^	NA	NA	NA	2 (7%)	3 (13%)	
Days in ICU (median (IQR))	3 (2, 8)	3 (2, 8)	0.957	6 (3, 15)	10 (3, 13)	0.938
SARS-CoV-2 tested^b^ (*n* (%))	0 (0%)	11 (32%)	NA	3 (11%)	10 (42%)	NA
SARS-CoV-2 positive^c^ (*n* (%))	NA	0 (0%)	NA	0 (0%)	0 (0%)	NA

Early interval includes weeks 1 to 11, and late interval includes weeks 12 to 22

Abbreviation: *GCS*, Glasgow Coma Scale; *TBI*, traumatic brain injury; *SAH*, aneurysmal subarachnoid hemorrhage

^a^In all cases aneurysm treatment was withheld due to dismal prognosis

^b^Tested during the initial admission

^c^Of SARS-CoV-2 tested

### Admissions and operations

**Fig. 1:**
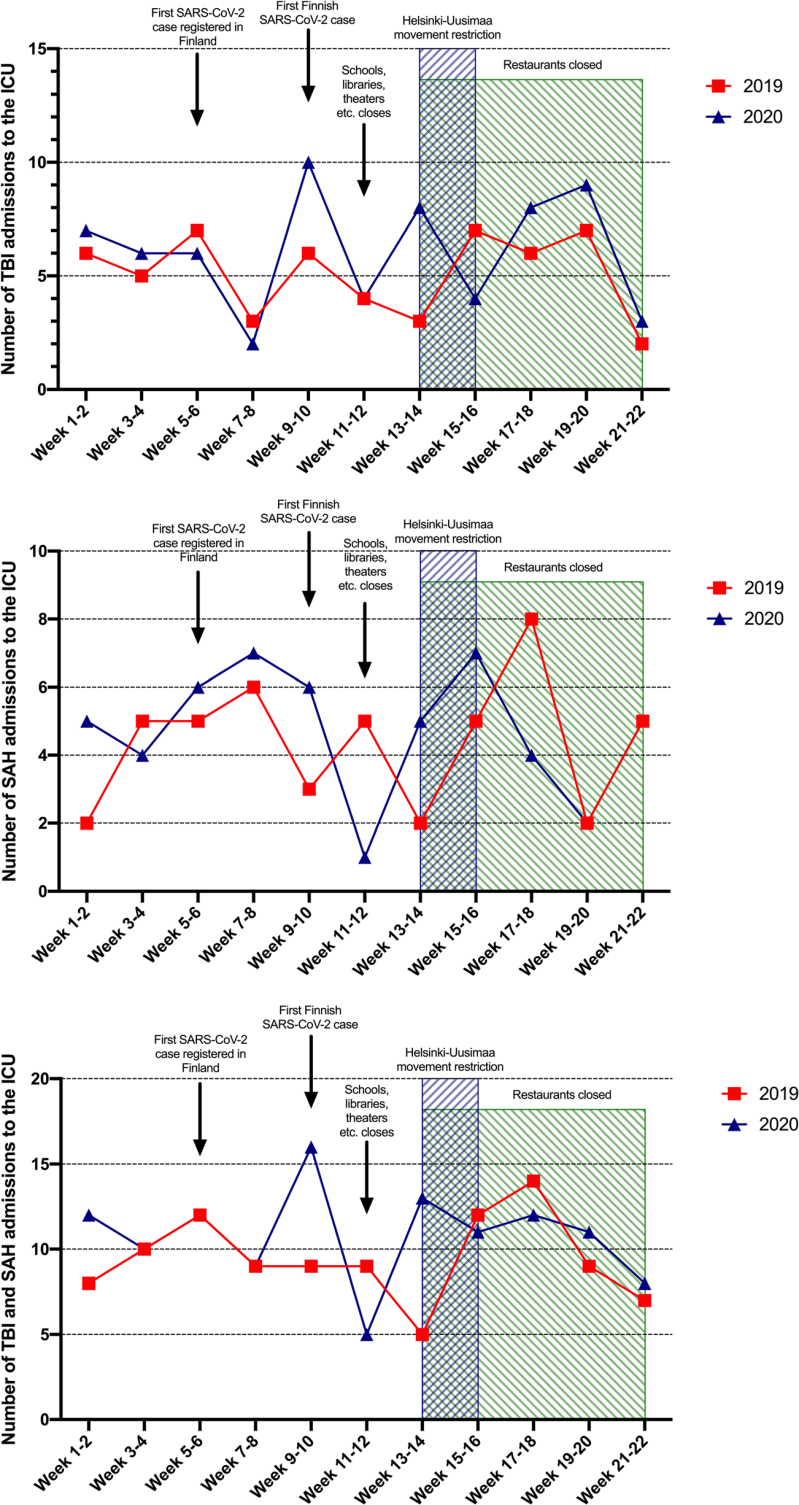
Number of TBI (upper), SAH (middle), and combined (lower) admissions to the neurosurgical ICU on a biweekly basis. Relevant Covid-19 pandemic features shown

**Fig. 2:**
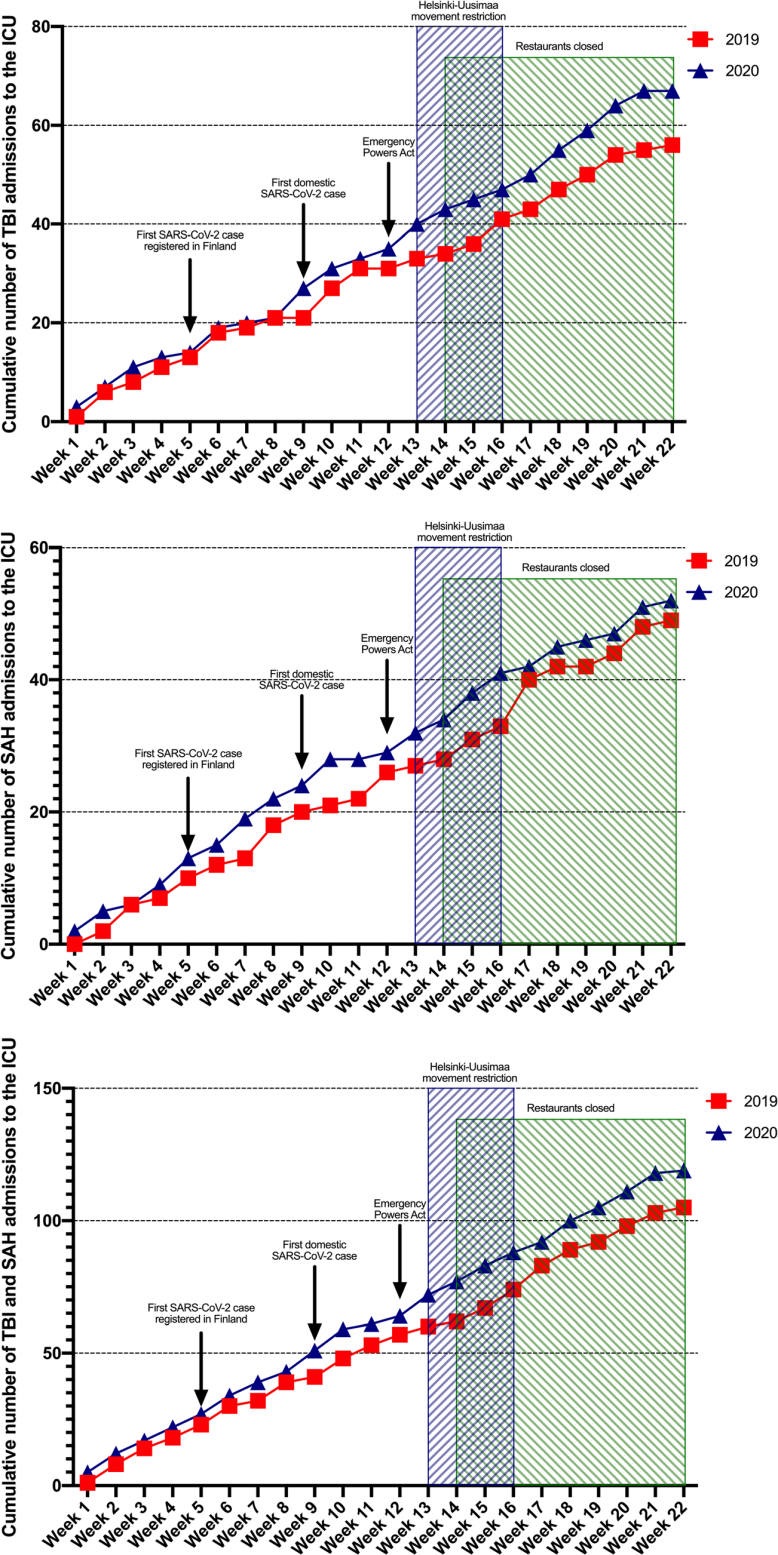
Cumulative number of patients with TBI (upper), SAH (middle), and combined (lower) admitted to the neurosurgical ICU. Relevant Covid-19 pandemic features shown

In comparison with 2019, no major changes in the number of admissions were seen in 2020 on a biweekly basis (Fig. 1). Inspection of the cumulative number of admissions revealed a minor difference between 2019 and 2020, indicating a minimal increase in 2020 (Fig. 2).

**Fig. 3:**
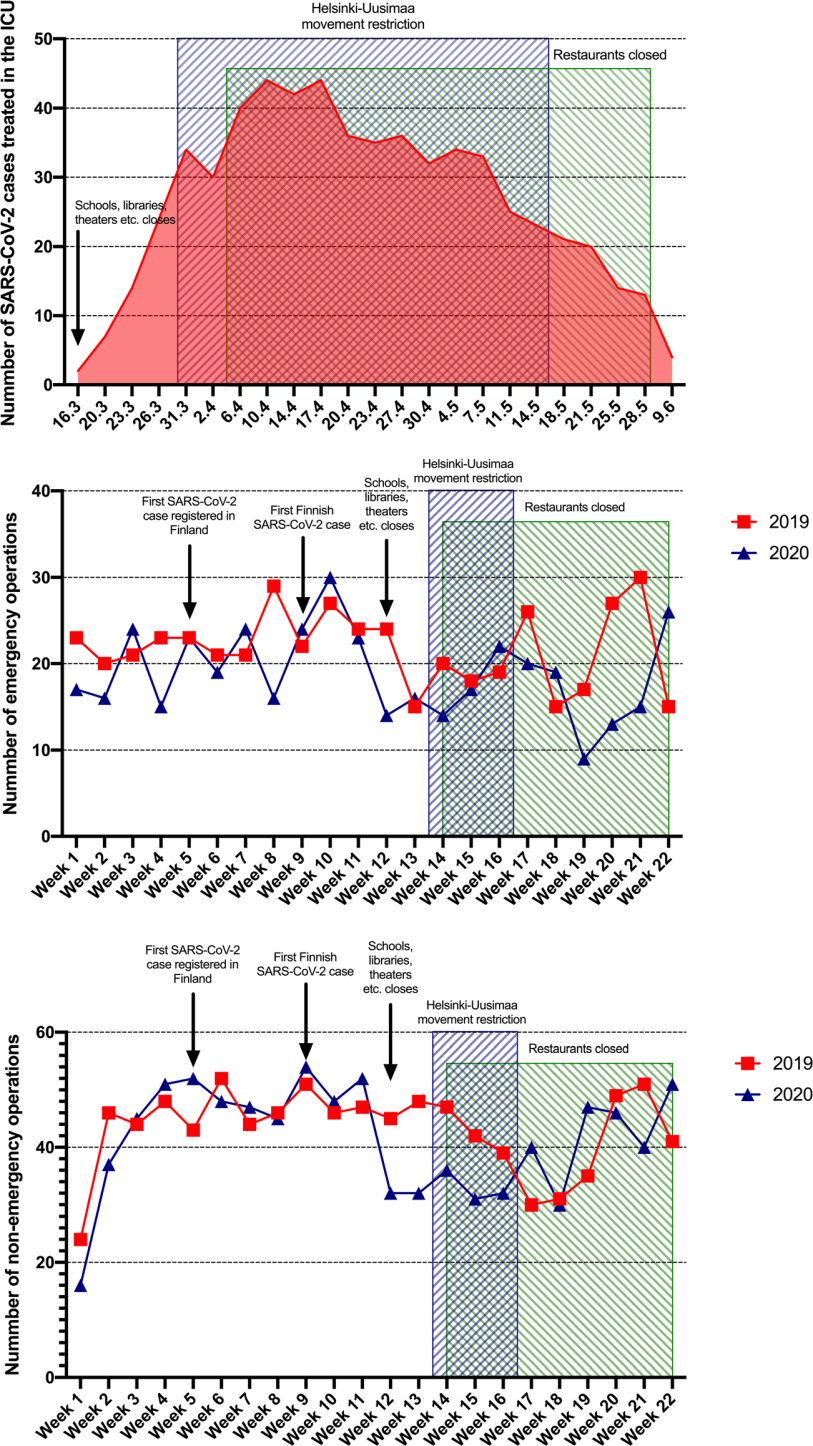
Number of patients with SARS-CoV-2 treated in intensive care units in the Helsinki and Uusimaa area (upper), number of emergency neurosurgical operations (middle), and number of non-emergency neurosurgical operations (lower). Operations shown for 2019 (red) and 2020 (blue). Relevant Covid-19 pandemic features shown

The number of emergency operations decreased after week 10 (Fig. 3), concurrent with the peak in the number of ICU-treated SARS-CoV-2 patients in Helsinki. Simultaneously, a major decrease in elective surgeries was noted.

### Mortality

**Fig. 4:**
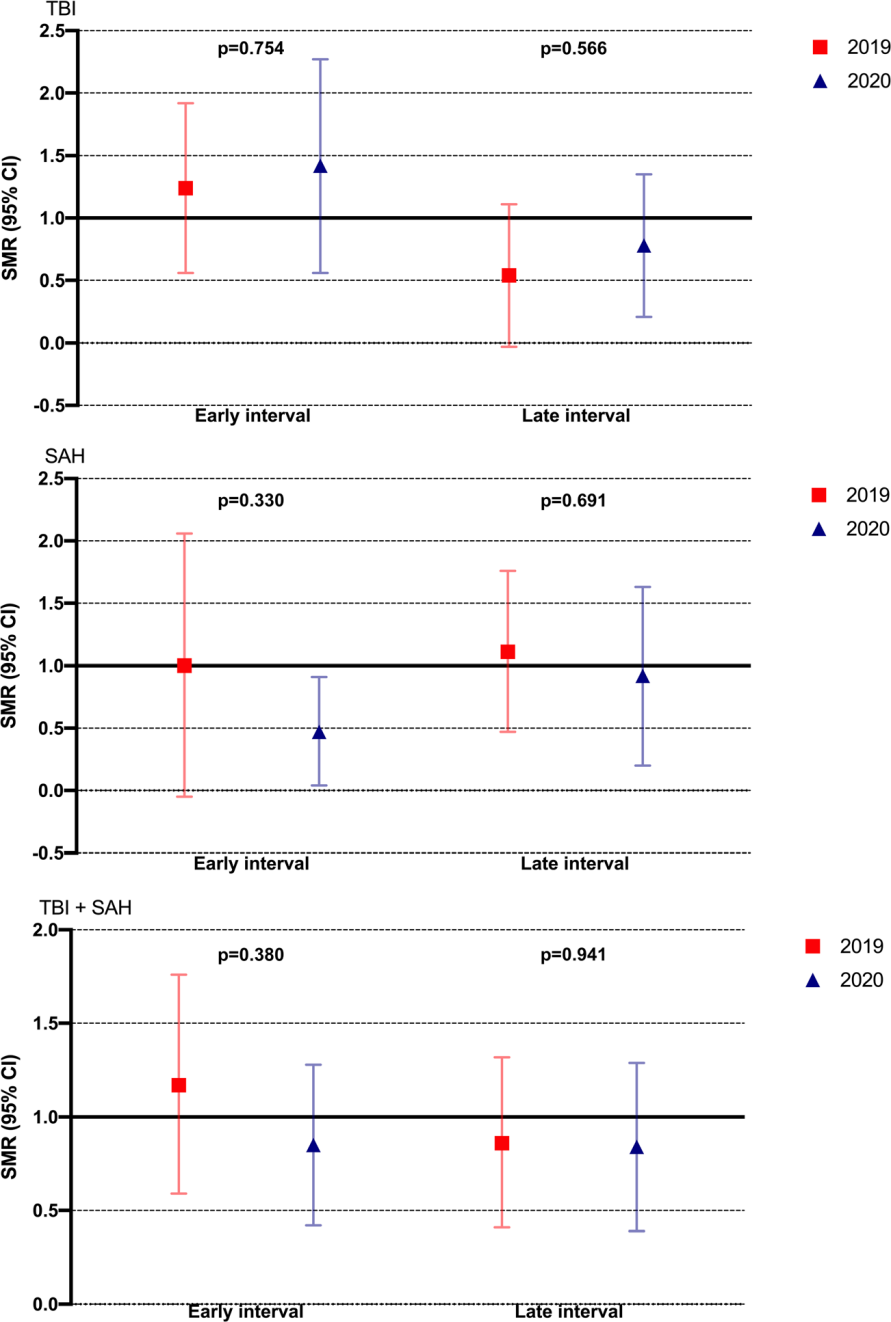
Standardized mortality rates (SMR) with 95% CI according to time interval for patients with traumatic brain injury (upper), subarachnoid hemorrhage (middle), and combined (lower). SMRs are calculated as 30-day mortality. The expected risk of 30-day mortality is adjusted for age, Glasgow Coma Scale score on admission, and pupillary light reactivity. SMRs are compared using a *t* test

The AUC of the predicted risk of death model was 0.88 (95% CI, 0.82–0.94), including all TBI and SAH patients admitted in 2020. For TBI patients admitted in 2020, the AUC was 0.85 (95% CI, 0.76–0.94), and in SAH patients admitted in 2020, the AUC was 0.90 (95% CI, 0.81–0.98). There was no notable difference in SMRs (Fig. 4) for patients treated in the early and late intervals in 2020 compared with 2019 for any of the patient groups (*p* > 0.05 for all comparisons).

## Discussion

We found that the number of TBI and aneurysmal SAH patients admitted to the neuro-ICU stayed the same during the Covid-19 pandemic compared with the previous year. Furthermore, we did not see any increase in risk of death related to TBI or SAH. The number of emergency and non-emergency neurosurgical operations was lower during the Covid-19 outbreak compared with the same timeframe in 2019.

Although the first SARS-CoV-2 infection was reported in Finland in January 2020 when a foreign tourist tested positive, it was not until March 2020 that the number of positive-tested SARS-CoV-2 patients began to rapidly increase. The first domestic SARS-CoV-2 case was on 26 February 2020, having contracted the infection from northern Italy. We began seeing a notable increase in the number of patients requiring intensive care due to SARS-CoV-2 in the beginning of March with a peak reached soon after the Uusimaa province’s lockdown and closing of restaurants in early April.

Thus, the first Covid-19 wave hit Finland later than some other countries in Europe, providing extra time to prepare the healthcare system. The operative plan and required procedures to increase ICU capacity were performed efficiently and promptly, which guaranteed sufficient ICU capacity throughout the Covid-19 outbreak, not only to treat SARS-CoV-2 patients but also to treat other patients requiring intensive care. Regarding neurosurgical care in Helsinki, the number of neuro-ICU beds and the number of elective neurosurgery were reduced and specialized neuro-ICU nurses were reallocated to Covid-19-specific ICUs. Still, it was decided that the neuro-ICU was only to treat Covid-19 patients if all other resources have been used. This decision ensured adequate priority and resources to care for emergency neurosurgical patients.

Reductions in elective neurosurgery have been reported throughout Europe, resulting in prolonged waiting times for elective surgery [9, 11]. As reported by Mathiesen et al., large variations in the magnitude of resource reallocation (neuro-ICU, capacity, ORs, beds) throughout Europe were noted [11]. Still, it seems that most of the units across Europe have been able to provide adequate treatment for neurosurgical emergency patients. In Helsinki, we did not end up in a situation where patient flow (of SARS-CoV-2 and non-SARS-CoV-2 patients) would have pushed the limits of our treatment capacity. Thus, any out of the normal ethical triage was not needed [8].

Interestingly, none of the TBI and aneurysmal SAH patients tested positive during the study period nor were there any known cases of TBI or aneurysmal that would not have been admitted due to SARS-CoV-2. Of note is that the local testing policy was that unconscious patients, in whom a of history SARS-CoV-2 symptoms could not be ruled out, were tested and isolated until negative results.

While the focus was to secure sufficient healthcare capacity to treat Covid-19 patients, there were concerns that the diagnosis and treatment of other acute diseases might suffer. Possible reasons for this could include a higher threshold for contacting healthcare services among the general population due to fear of contracting Covid-19 or acute diseases being left unnoticed due to the effects of social distancing [16]. In fact, hospital admissions due to transient ischemic attack (TIA), acute stroke, and acute coronary syndrome decreased during the Covid-19 pandemic [3, 4, 6]. Also, one report indicates that time from stroke onset to hospital arrival was prolonged during the pandemic [17]. However, in contrast with TIA or milder forms of ischemic stroke, the symptoms of aneurysmal SAH are often so severe that delaying contact with healthcare seems unlikely. According to our hypothesis, we found that the number of neuro-ICU-admitted aneurysmal SAH stayed largely the same in 2019 and 2020. In retrospect, one could have argued that the aneurysmal SAH incidence would decrease due to the collapse in incidence of the normal seasonal influenza epidemic [18] as it has been speculated that respiratory infections might predispose aneurysm rupture [2, 14]. In fact, in a large European survey study, 13 out of 25 centers reported a lower number of ICU-treated SAH patients during the Covid-19 pandemic [11]. Whether this is due to the lower rate of seasonal influenza or due to other reasons remains open.

In contrast with our hypothesis, we did not see a decline in ICU admissions due to TBI during the heavy restrictions imposed in Finland. For example, in the European survey study, 18 out of 25 centers reported a lower number of ICU-treated TBI patients. Thus, our finding was surprising, in part because one would assume that self-isolation and closing of bars would prevent head injuries, considering that over a third of TBIs in Finland occur under the influence of alcohol [12]. Yet, the closing of restaurants and bars may have just shifted the place of alcohol consumption, as alcohol sales reported by the state-owned Alko Inc. (the national alcoholic beverage retail monopoly in Finland, controlling the trade and sale of alcoholic beverages > 5.5%) increased [1]. This seems plausible considering that half to two-thirds of all TBIs treated in the neuro-ICU are low-energy fall [13]. For example, the number of people injured in traffic accidents during January to May 2020 decreased by 25% compared with the same period in 2019, although the number of deaths due to traffic accidents remained unchanged (data from Statistics Finland) [15]. Thus, it seems there was a reduction in the number of milder injuries due to the lockdown, but the number of severe injuries stayed the same.

Similar to our findings, Hecht and coworkers from Berlin reported a decline in all neuro-emergencies at the Charité University Hospital during the Covid-19 pandemic. They specifically reported a no change in the number of aneurysmal SAH and TBI admissions [5].

Although the restrictions did not affect the rate of ICU admissions, we had temporarily fewer emergency operations during the lockdown. Also, the number of emergency craniotomies for TBI declined between the early and late intervals of 2020. However, an overall reduction in emergency operations was also seen in 2019 and may be related to normal seasonal variation instead of Covid-19. As expected, the rate of elective operations declined following the decision to cut non-urgent treatment.

## Conclusions

The strict nationwide restrictions enforced in Finland due to the Covid-19 pandemic did not result in a notable drop in the number of neuro-ICU-treated TBI or aneurysmal SAH patients. We did not find evidence of worse prognoses in TBI and SAH patients during the Covid-19 pandemic.
